# Supplementary material for: A peptide encoded by circular form of LINC-PINT suppresses oncogenic transcriptional elongation in glioblastoma
Source: Nat Commun. 2018 Oct 26;9:4475. doi: 10.1038/s41467-018-06862-2 (PMC6203777; doi:10.1038/s41467-018-06862-2)
Supplement: Supplementary file 1 — Supplementary Data 2 [file 41467_2018_6862_MOESM1_ESM.pdf]

GenScript
Make Research Easy

One Stop Solution for Biology Research

Certificate of Analysis

CONFIDENTIAL

SC1180 Express Complete Affinity-Purified Peptide Polyclonal Antibody Package

Order ID: 7142636-1

Report From:

GenScript USA Inc

860 Centennial Ave

Piscataway, NJ 08854

United States of America

For research use only

CONFIDENTIAL

GenScript
Make Research Easy

One Stop Solution for Biology Research

1. Project Information

Order ID: 7142636-1
Antigen Name: 1
Sequence: CGEESIRGAHGYKNK (Lot: 7142638-1)
Immunogen: Peptide-KLH conjugate
Host Strain: New Zealand Rabbit

2. Product Information(s)

Product 1: Antigen 7142638-1
Form: Lyophilized
Quantity: Total 2.00 mg in one vial

Product 2: Pre-immune serum
Form: Liquid
Preservative: 0.02% Sodium Azide
Volume: 0.50 ml/vial
Quantity: One vial

Product 3: Affinity-Purified antibody
Form: Liquid
Concentration: 1.828 mg/ml (by A280nm)
Purity: 91% (by SDS-PAGE)
Quantity: Total 7.31 mg in one bottle
Buffer: Phosphate Buffered Saline (PBS, pH 7.4) with 0.02% Sodium Azide.

CONFIDENTIAL

3. QC Results

Indirect ELISA:

Coating Antigen(s): Free Peptide
Coating Concentration: 4 µg/ml, 100 µl/well
Coating Buffer: Phosphate Buffered Saline, pH7.4
Secondary Antibody: Anti-RABBIT IgG (H&L) (GOAT) Antibody Peroxidase Conjugated (Min X Bv Ch Gt GP Ham Hs Hu Ms Rt & Sh Serum Proteins)

Table 1. Elisa results of Pre-immune serum and Purified antibody:

	Dilution	Anti-1 Antibody
NC	1:1,000	0.067
1	1:1,000	2.735
2	1:2,000	2.720
3	1:4,000	2.676
4	1:8,000	2.467
5	1:16,000	2.344
6	1:32,000	2.150
7	1:64,000	1.773
8	1:128,000	1.293
9	1:256,000	0.846
10	1:512,000	0.559
11	Blank	0.051
12	Blank	0.051
	Titer	>1:512,000

Starting dilution: 1:1,000 (Equivalent to 1 µg/ml)
The titer is the highest dilution with S/B (Signal/Blank) >=2.1
NC is negative control (Pre-immune serum)

Approved by

David Chang

Director, Antibody Department

GenScript USA Inc

CONFIDENTIAL
