# Supplementary material for: A peptide encoded by circular form of LINC-PINT suppresses oncogenic transcriptional elongation in glioblastoma
Source: Nat Commun. 2018 Oct 26;9:4475. doi: 10.1038/s41467-018-06862-2 (PMC6203777; doi:10.1038/s41467-018-06862-2)
Supplement: Supplementary file 2 — Supplementary Information [file 41467_2018_6862_MOESM2_ESM.pdf]

Cover Page

Zhang *et al*. **A Peptide Encoded by Circular Form of *LINC-PINT* Suppresses Oncogenic Transcriptional Elongation in Glioblastoma.**

a

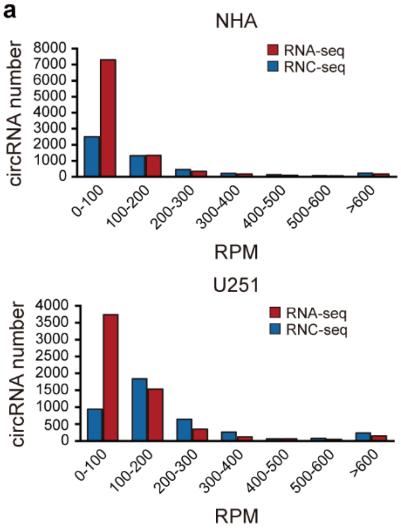

b

CircBase

ID	hsa_circ_0082389
Position	chr7:130792478-130793562
Strand	—
Genomic length	1084
Spliced sequence length	1084
Annotation	ANNOTATED ncRNA OVEXON
Repeats	DNA
Best transcript	NR_015431
Gene symbol	FLJ43663

Supplementary Fig. 1 Identification of *circPINTexon2* in human Circbase.

a Identified circRNA numbers by using RNA-seq and RNC-seq in NHA and U251 cells. **b** Annotated *circPINTexon2* in circBase.

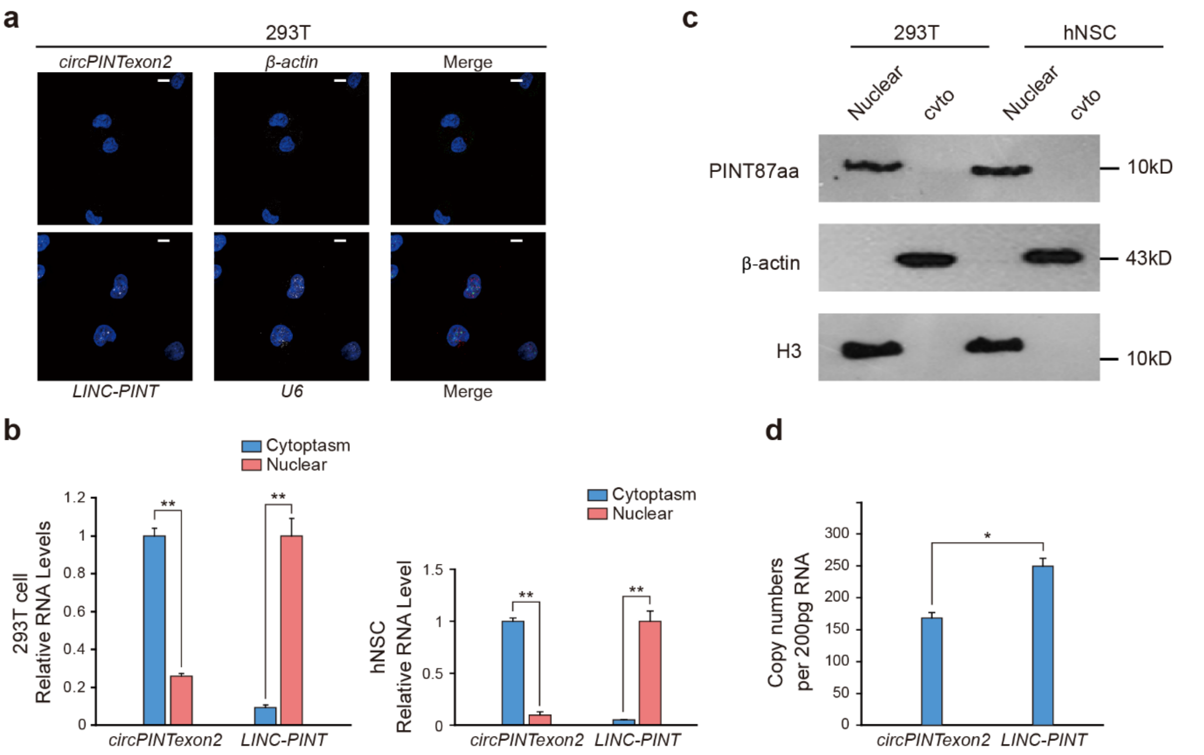

Supplementary Fig. 2 Localization of *circPINTexon2* and *LINC-PINT*.

a *circPINTexon2* and *LINC-PINT* were detected via double-stained FISH in 293T cells with specific probes. *β-actin* and *U6* mRNA were used as cytoplasmic and nuclear markers, respectively. Scale bar, 20 μM. **b** Nuclear and cytoplasmic fractions from 293T cells were isolated with a PARIS™ Kit (Thermo Fisher), and total RNA was extracted. q-PCR was performed to test the expression of *circPINTexon2* and linear *LINC-PINT* in different cell fractions, as indicated. **c** Nuclear and cytoplasmic fractions from 293T cells and hNSC were isolated with a PARIS™ Kit (Thermo Fisher), and total protein was extracted. PINT87aa expression was determined by western blot. **d** Copy numbers of *circPINTexon2* and *LINC-PINT* in 200pg RNA was determined by absolute q-PCR in 293T cells. The standard curve was generated by serial dilution. **b**, **d** Data are resented as mean ± s.e.m. from three independent experiments. *$P < 0.05$; ns, $P > 0.05$, determined by two-tailed Student's *t*-tests.

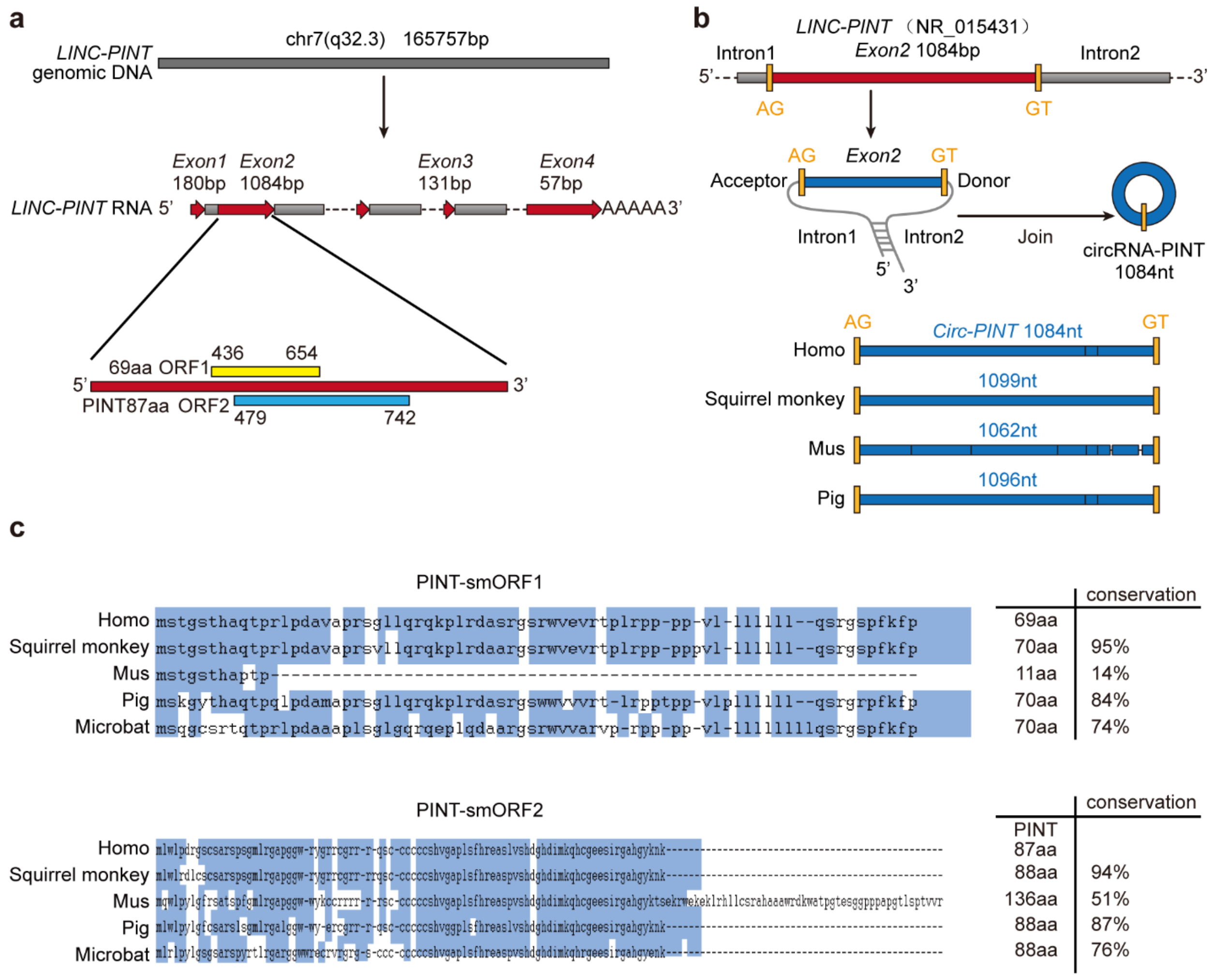

Supplementary Fig. 3 Conservation analysis of putative ORFs in *LINC-PINT* exon 2.

a The locations of putative ORFs encoding the 69-aa peptide and 87-aa peptide in *LINC-PINT* exon 2 are shown. **b** Putative circulating strategy for the *LINC-PINT* exon 2 in different species. **c** Conservation of putative ORFs encoding the 69-aa peptide and 87-aa peptide in *LINC-PINT* exon 2 among different species. The 87-aa peptide demonstrates better conservation than the 69-aa peptide.

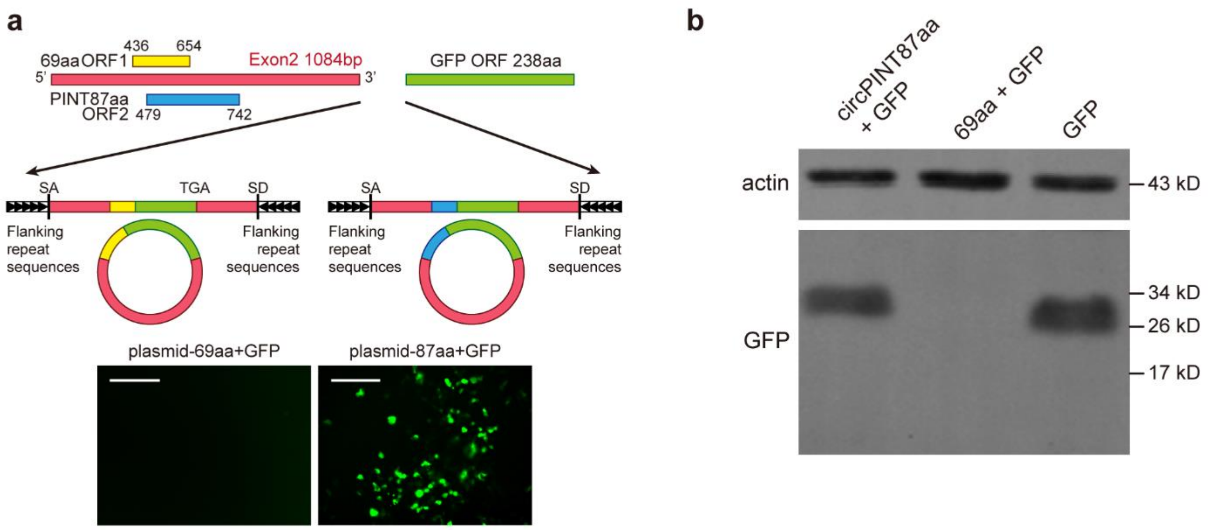

Supplementary Fig. 4 Identification of activated ORFs in *LINC-PINT* exon 2.

a Illustration of plasmid construction. The sequence encoding GFP was cloned immediately after the 69-aa or 87-aa putative ORF in *LINC-PINT* exon 2 after deletion of the ATG start codon and the TAA stop codon. The modified whole *LINC-PINT* exon 2 was then cloned into the pSin-EF2CMV-puro expression vector and transfected into HEK293T cells. At 48 h after transfection, only the 87-aa-GFP conjugated protein was expressed. A plasmid containing only GFP was used as a positive control. Note that although this linear vector translated the 87-aa ORF, the vector was artificially constructed (only exon 2 of *LINC-PINT* was cloned) and driven by the CMV promoter. Overexpression of this CMV-driven plasmid in HEK293T cells could theoretically translate any in-frame active ORF. These results did not show that full-length linear LINC-PINT was translated from the 87-aa ORF *in vivo*. **b** Immunoblotting indicated that the 87-aa-GFP conjugated protein was detectable in HEK293T cells with a GFP antibody. Scale bar, 100 μM.

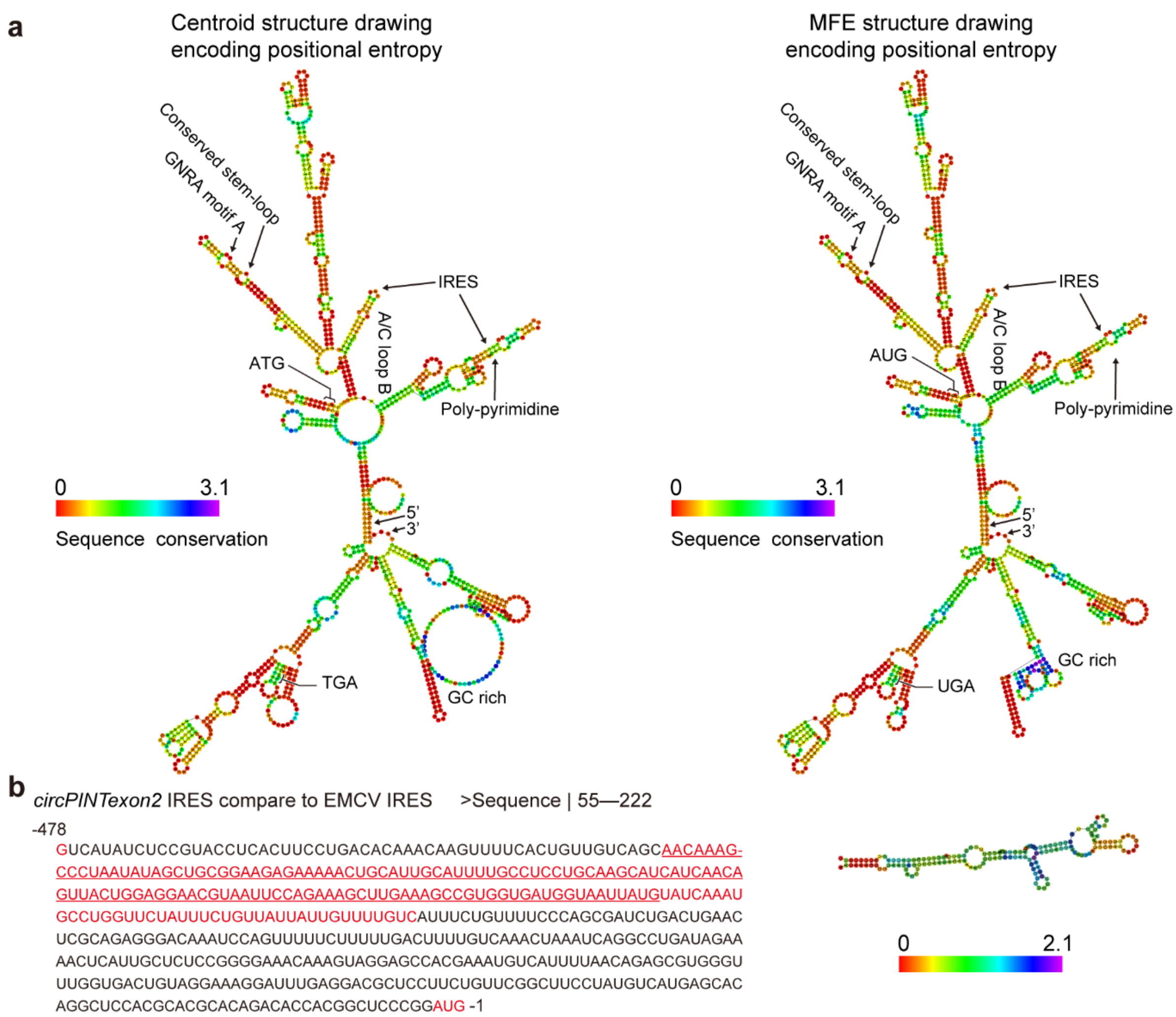

Supplementary Fig. 5 The predicted secondary structure of *LINC-PINT* exon 2 and the putative IRES site.

a The secondary structure of *LINC-PINT* exon 2 was predicted according to http://rna.tbi.univie.ac.at, and the putative IRES was determined according to http://www.iresite.org. Mammalian IRES usually contains the conserved GNRA stem-loop A, an A/C-rich loop B, and a poly-pyrimidine region. R represents A/G, and N is a random nucleotide.

b Species conservation analysis of the *circPINTexon2* IRES and EMCV IRES.

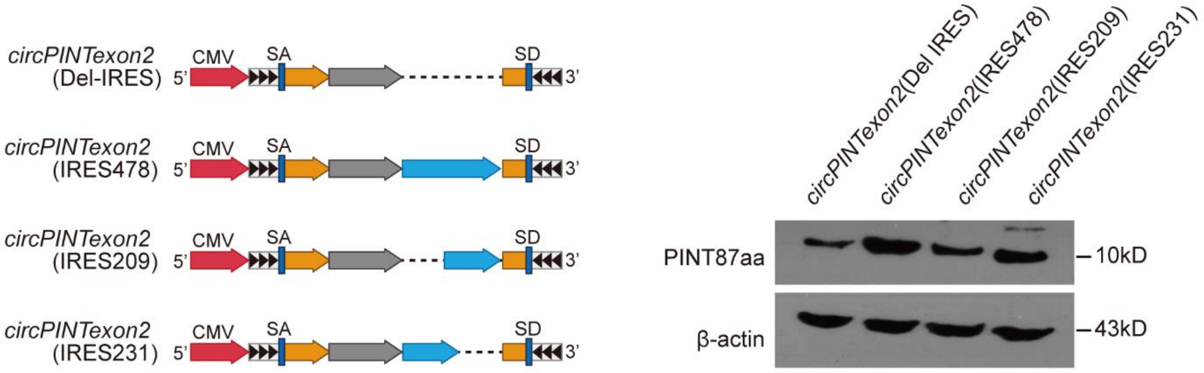

Supplementary Fig. 6 IRES activity was tested in artificial circular plasmids.

Schematic illustrations showing the artificial plasmids containing different truncations of the *circPINTexon2* IRES. These plasmids were transfected in to HEK293T cells, and PINT87aa expression was tested.

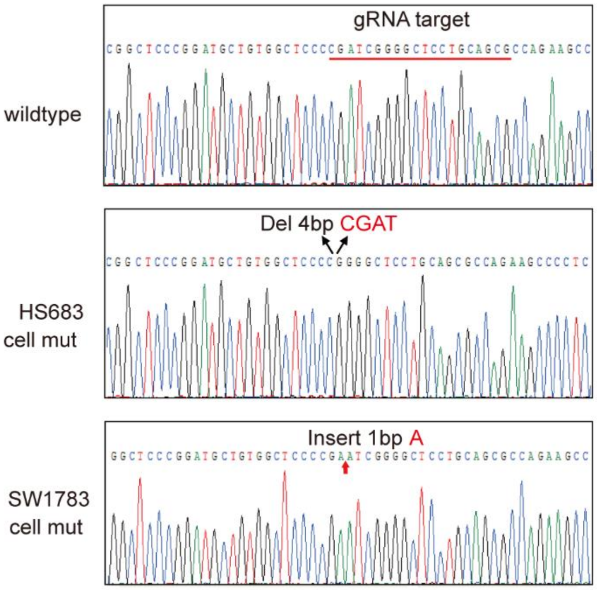

Supplementary Fig. 7 Construction of PINT87aa ORF deletion Hs683 and SW1783 cells using CRISPR/Cas9.

A small fragment inside the 87-aa ORF was deleted using the CRISPR/Cas9 system. Sanger sequencing confirmed a 4-bp deletion in Hs683 cells and a 1-bp insertion in SW1783 cells, both of which interrupted PINT87aa ORF translation.

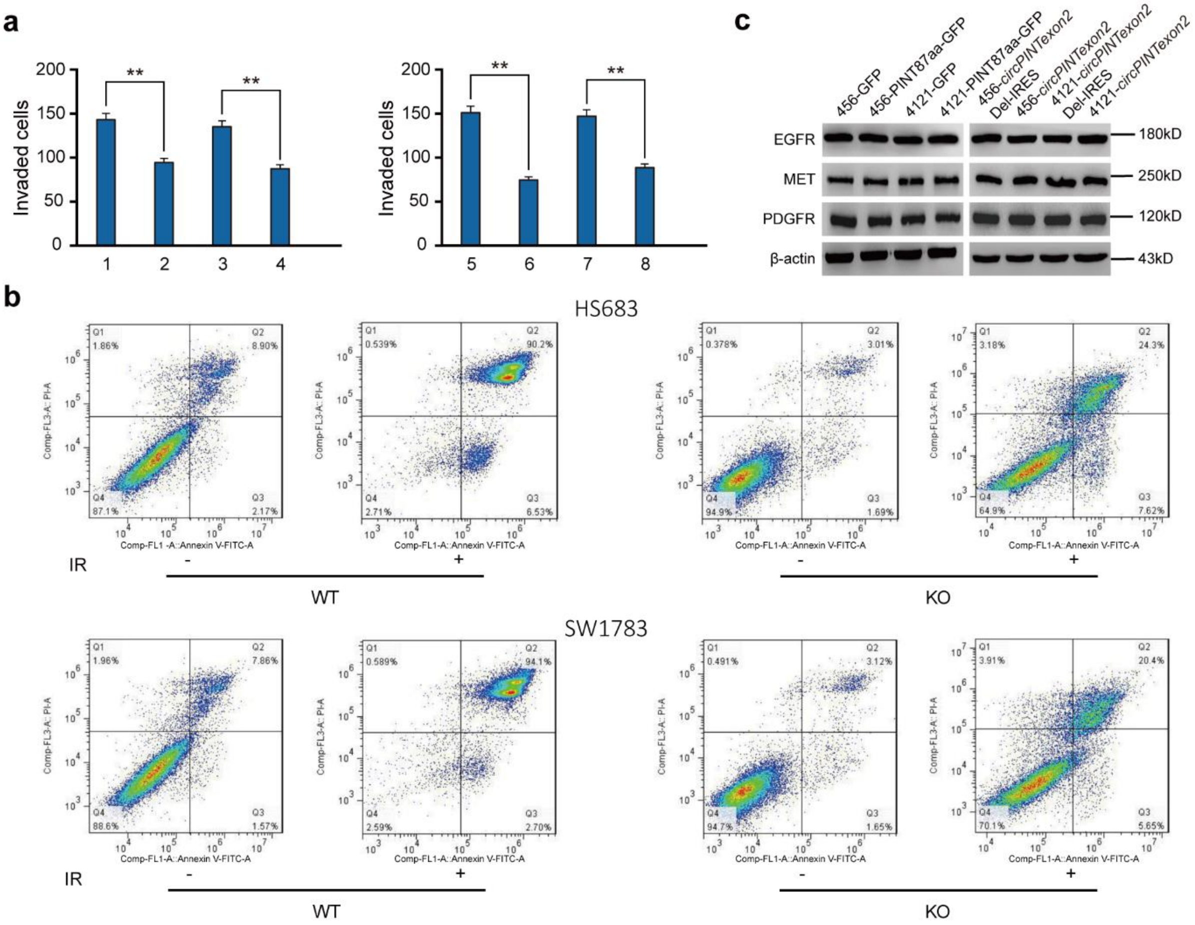

Supplementary Fig. 8 PINT87aa biological function.

a Transwell assay *in vitro*. 1. 456-GFP, 2. 456-PINT87aa, 3. 4121-GFP, 4. 4121-PINT87aa, 5. 456-*circPINTexon2* IRES, 6. 456-*circPINTexon2*, 7. 4121-*circPINTexon2* IRES, 8. 4121-*circPINTexon2* IRES. Data are presented as mean ± s.e.m. from three independent experiments. *$P < 0.05$; ns, $P > 0.05$, determined by two-tailed Student's *t*-tests. **b** PINT87aa K.O. SW1783 and Hs683 cells and the control cells were subjected to 6 Gy radiation. DNA damage was determined by flow cytometry and γ-H2X expression. At least three independent experiments were repeated. **c** *EGFR*, *MET*, *PDFGR* level were determined in PINT87aa or *circPINTexon2* stably transduced 456 and 4121 cells and their control cells.

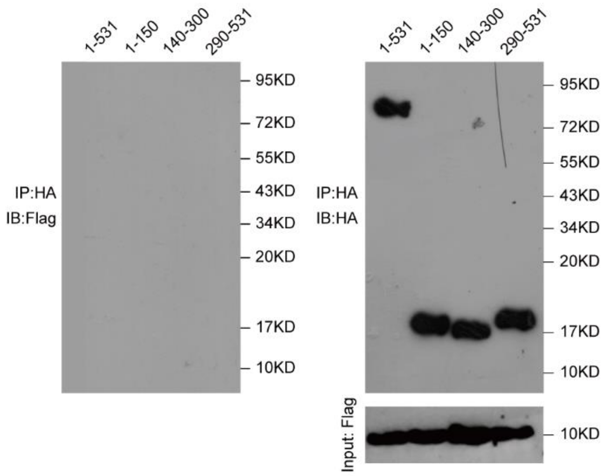

Supplementary Fig. 9 Determine the PAF1 binding site in PINT87aa.

Flag-tagged R20, G21, P23, C32, R36 and S53 mutation PINT87aa was co-transfected with HA-PAF1 in 293T cells. Immunoprecipitation was performed by using HA-antibody and following western blot was performed by using HA and Flag antibody.

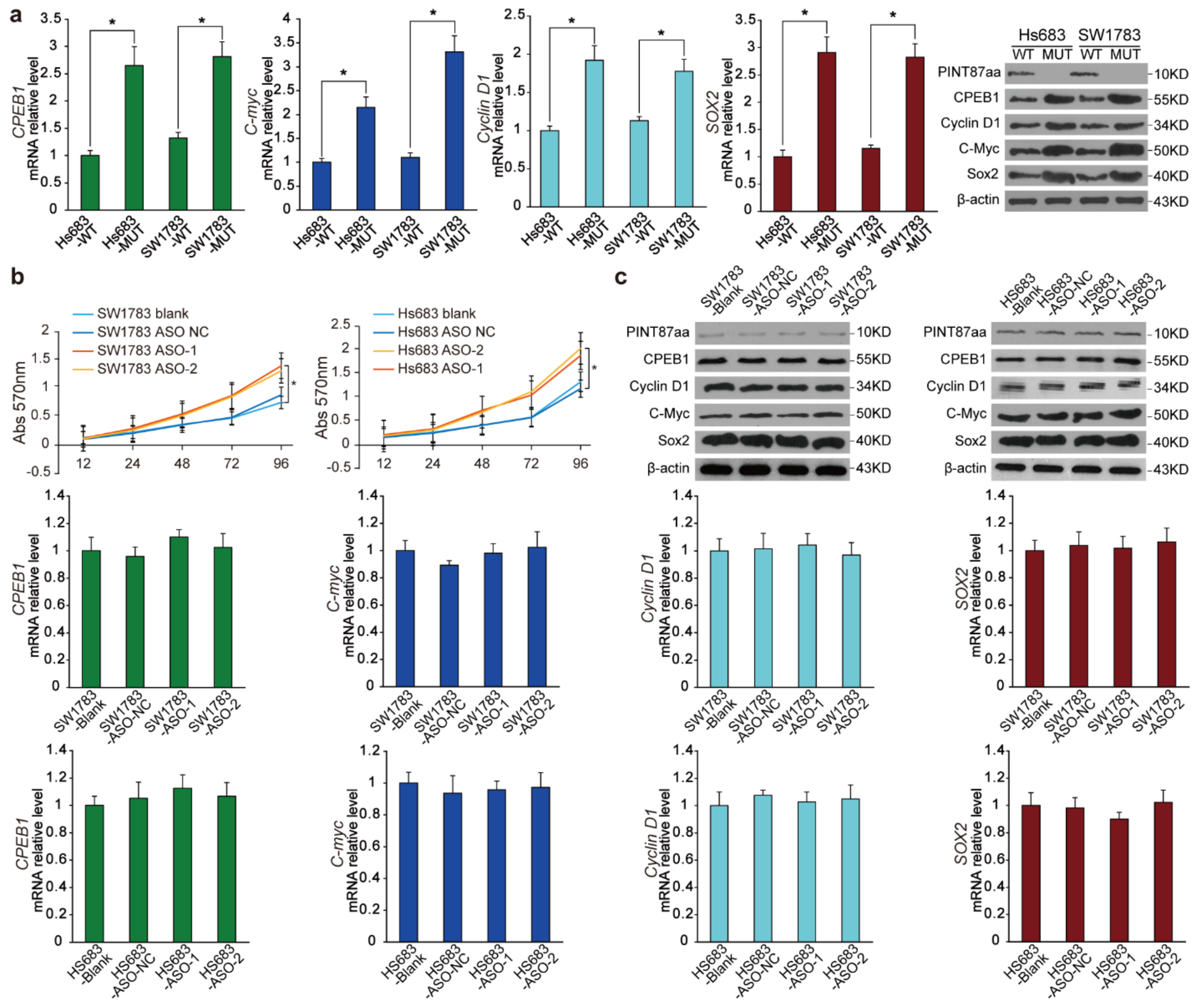

Supplementary Fig. 10 PAF1 downstream targets were transcriptionally regulated by PIN87aa.

a *CPEB1*, *cyclin D1*, *c-Myc* and *SOX-2* mRNA and protein levels were tested in PINT87aa K.O. Hs683 and SW1783 cells. **b** Cell proliferation rate was determined in PINT87aa K.O. Hs683 and SW1783 cells. **c** Two specific ASOs designed to target linear *LINC-PINT* were transfected into Hs683 and SW1783 cells. *CPEB1*, *cyclin D1*, *c-Myc* and *SOX-2* mRNA and protein levels were tested at 48 h after transfection. **a, b, c** Data are presented as mean ± s.e.m. from three independent experiments. **$P < 0.01$; *$P < 0.05$; ns, $P > 0.05$, determined by two-tailed Student's *t*-tests.

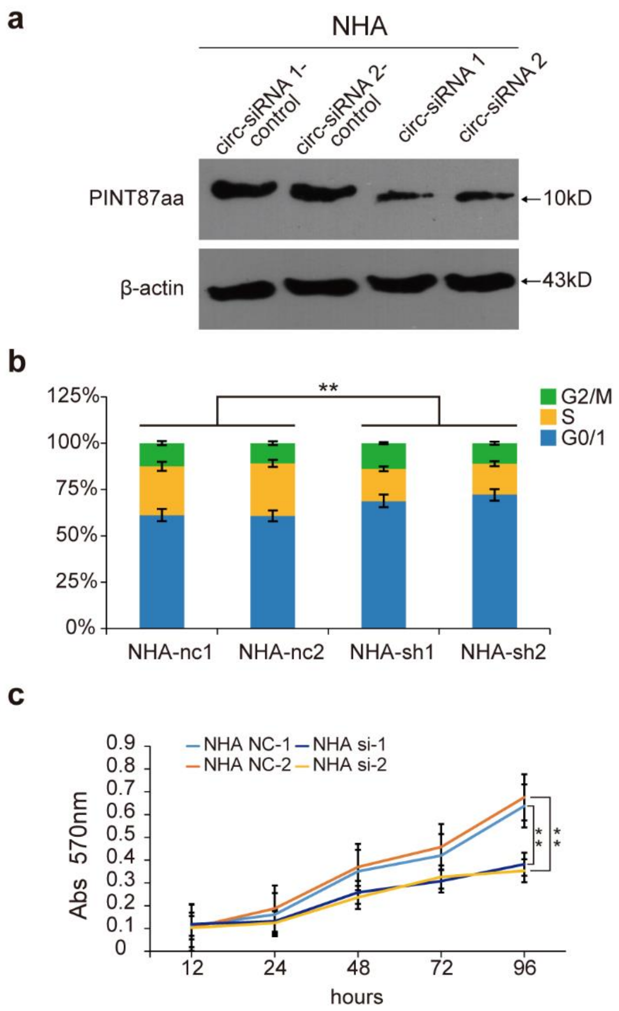

Supplementary Fig. 11 Impact of altered PINT87aa expression on cell growth *in vitro*.

a Stable knockdown of PINT87aa in NHA cells using two independent shRNAs. **b** PINT87aa-deprived NHA cells exhibited a decrease in S phase and an increase in G0/1 phase. **c** NHA cells with stable PINT87aa knockdown showed a lower proliferation rate. **b, c** Data are presented as mean ± s.e.m. from three independent experiments. ***P* < 0.01; ns, *P*> 0.05, determined by two-tailed Student's *t*-tests

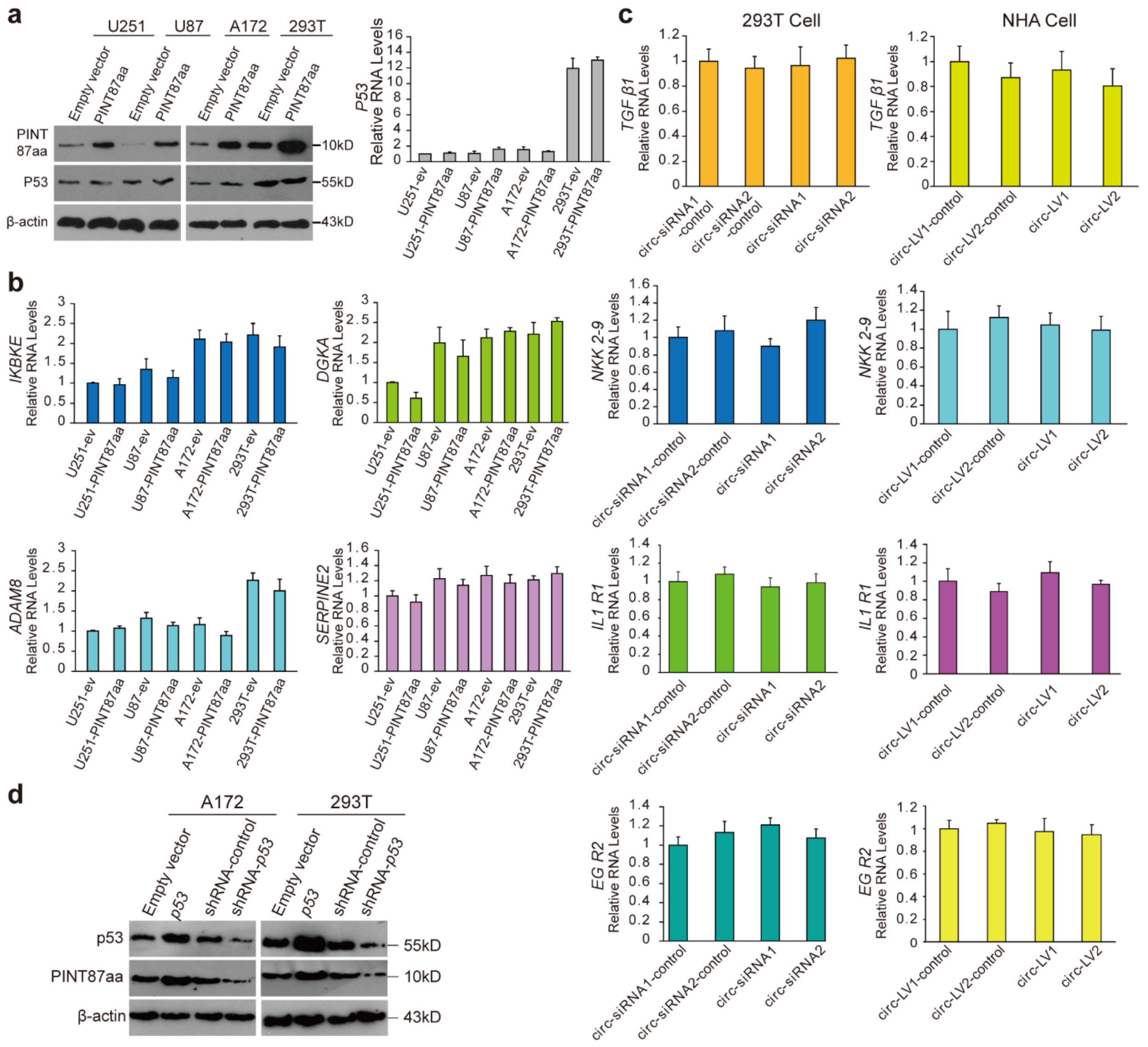

Supplementary Fig. 12 ***p53*** **was detected in** ***circPINTexon2*** **circRNA-overexpressing cells.**

a *CircPINTexon2* overexpression induced PINT87aa up-regulation, but p53 status did not change. **b** *LINC-PINT* downstream genes *IKBKE*, *DGKA*, *ADAM8* and *SERPINE2* were not altered after *circPINTexon2* transfection. **c** *LINC-PINT* downstream genes *TGFB1*, *NKX2-9*, *IL1R1*, and *EGR2*, were detected by q-PCR in *circPINTexon2*-specific siRNA-transfected 293T and NHA cells. **b, c** $^{**}P < 0.01$; ns, $P > 0.05$, determined by two-tailed Student's *t*-tests. **d** *P53* was stably transduced in A172 and 293T cells. PINT87aa expression was determined.

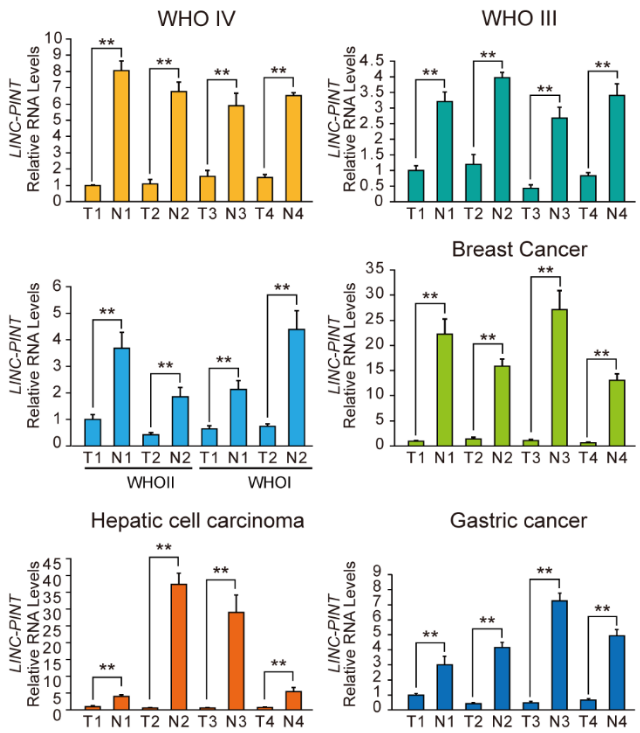

Supplementary Fig. 13 Linear *LINC-PINT* expression in several human malignancies and adjacent normal tissues.

Several paired human cancer tissues and adjacent normal tissues (the same tissues shown in Fig. 4) were subjected to q-PCR analysis using linear *LINC-PINT*-specific primers. $^{**}P < 0.01$; ns, $P > 0.05$, determined by two-tailed Student's *t*-tests

Supplementary Table 1. Annotation rate of circRNAs from circBase.

**CircBase**	**CircRNA number**	**CircRNA recorded in circBase**	**Annotation rate from circBase**
RNA-seq	7,016	3,303	47.08%
RNC-seq	12,863	3,975	30.90%
RNA-seq (average RPM>100)	2,448	1,542	62.99%
RNC-seq (average RPM>100)	1,586	983	61.98%
RNA-seq (average RPM<=100)	4,568	1,761	38.55%
RNC-seq (average RPM<=100)	11,277	2,992	26.53%

Supplementary Table 2. Sequencing depth of RNA-seq and RNC-seq.

**Sample**	**Clean Data(bp)**	**Q20(%)**	**Q30(%)**
NHA-RNC	71,365,714,950	69,469,221,718 (97.34%)	66,051,111,845 (92.55%)
NHA-RNA	17,639,669,924	17,227,387,816 (97.66%)	16,488,445,529 (93.47%)
U251-RNC	86,980,594,910	84,672,062,021 (97.35%)	80,464,669,535 (92.51%)
U251-RNA	16,185,135,272	15,836,185,796 (97.84%)	15,200,074,412 (93.91%)

Supplementary Table 3. GO analysis of enriched host genes.

GO ID	Description	GeneRatio (250)	BgRatio (19693)	P value	Fdr	Type
GO:0044424	intracellular part	221	14327	1.12E-09	6.91E-08	Cellular Component
GO:0005622	intracellular	225	14878	4.22E-09	1.31E-07	Cellular Component
GO:0043226	organelle	211	13705	4.23E-08	8.75E-07	Cellular Component
GO:0005654	nucleoplasm	69	3015	4.01E-07	6.21E-06	Cellular Component
GO:0043229	intracellular organelle	194	12431	5.38E-07	6.68E-06	Cellular Component
GO:0044422	organelle part	88	4339	1.17E-06	1.03E-05	Cellular Component
GO:0044446	intracellular organelle part	88	4339	1.17E-06	1.03E-05	Cellular Component
GO:0005737	cytoplasm	176	11132	3.92E-06	3.04E-05	Cellular Component
GO:0005829	cytosol	71	3400	7.68E-06	5.29E-05	Cellular Component
GO:0044428	nuclear part	78	3917	1.34E-05	8.28E-05	Cellular Component
GO:0031974	membrane-enclosed lumen	73	3639	2.25E-05	9.96E-05	Cellular Component
GO:0031981	nuclear lumen	73	3639	2.25E-05	9.96E-05	Cellular Component
GO:0043233	organelle lumen	73	3639	2.25E-05	9.96E-05	Cellular Component
GO:0070013	intracellular organelle lumen	73	3639	2.25E-05	9.96E-05	Cellular Component
GO:0044464	cell part	237	17225	7.86E-05	0.000324923	Cellular Component
GO:0005623	cell	238	17369	0.000104913	0.000387127	Cellular Component
GO:0005856	cytoskeleton	48	2187	0.000106148	0.000387127	Cellular Component
GO:0044444	cytoplasmic part	128	7842	0.000161157	0.000555096	Cellular Component
GO:0005634	nucleus	119	7187	0.000191835	0.000625987	Cellular Component
GO:0043228	non-membrane-bounded organelle	72	3863	0.000285227	0.000842097	Cellular Component
GO:0043232	intracellular non-membrane-bounded organelle	72	3863	0.000285227	0.000842097	Cellular Component
GO:0043227	membrane-bounded organelle	170	11358	0.000476827	0.001285359	Cellular Component
GO:0043231	intracellular membrane-bounded organelle	170	11358	0.000476827	0.001285359	Cellular Component
GO:0032991	macromolecular complex	86	5062	0.001304258	0.003369333	Cellular Component
GO:0005694	chromosome	23	918	0.001523385	0.003777994	Cellular Component
GO:0043234	protein complex	82	4820	0.001731284	0.004128447	Cellular Component
GO:0000228	nuclear chromosome	14	512	0.00602385	0.013832544	Cellular Component
GO:0005488	binding	167	10392	1.84E-06	7.80E-05	Molecular Function

GO:0005515	protein binding	68	3139	2.60E-06	7.80E-05	Molecular Function
GO:0016874	ligase activity	16	462	0.000263838	0.005276763	Molecular Function
GO:0003824	catalytic activity	90	5311	0.000728826	0.008744434	Molecular Function
GO:0019899	enzyme binding	38	1792	0.000981989	0.008744434	Molecular Function
GO:0016301	kinase activity	22	846	0.00106634	0.008744434	Molecular Function
GO:0043167	ion binding	106	6572	0.001165641	0.008744434	Molecular Function
GO:0008134	transcription factor binding	17	582	0.001165925	0.008744434	Molecular Function
GO:0016772	transferase activity, transferring phosphorus-containing groups	24	976	0.001362308	0.009082055	Molecular Function
GO:0000988	protein binding transcription factor activity	16	601	0.004036797	0.024220785	Molecular Function
GO:0019538	protein metabolic process	107	5032	1.20E-08	1.44E-06	Biological Process
GO:0043170	macromolecule metabolic process	121	6034	2.02E-08	1.44E-06	Biological Process
GO:0044267	cellular protein metabolic process	102	4805	3.96E-08	1.44E-06	Biological Process
GO:0006464	cellular protein modification process	90	4050	5.22E-08	1.44E-06	Biological Process
GO:0036211	protein modification process	90	4050	5.22E-08	1.44E-06	Biological Process
GO:0043412	macromolecule modification	90	4050	5.22E-08	1.44E-06	Biological Process
GO:0044260	cellular macromolecule metabolic process	116	5813	7.61E-08	1.80E-06	Biological Process
GO:0044238	primary metabolic process	136	7608	2.14E-06	3.71E-05	Biological Process
GO:0071704	organic substance metabolic process	136	7608	2.14E-06	3.71E-05	Biological Process
GO:0044237	cellular metabolic process	166	9925	2.23E-06	3.71E-05	Biological Process
GO:0008152	metabolic process	178	11093	9.98E-06	0.000150551	Biological Process
GO:0016043	cellular component organization	105	5707	3.23E-05	0.000443108	Biological Process
GO:0051276	chromosome organization	32	1128	3.47E-05	0.000443108	Biological Process
GO:0006996	organelle organization	62	2919	6.19E-05	0.000733447	Biological Process
GO:0071840	cellular component organization or biogenesis	105	5838	8.92E-05	0.000987278	Biological Process
GO:0000902	cell morphogenesis	34	1496	0.001245662	0.012163521	Biological Process
GO:0032989	cellular component morphogenesis	34	1496	0.001245662	0.012163521	Biological Process
GO:0040007	growth	24	980	0.002617997	0.024143754	Biological Process
GO:0007049	cell cycle	38	1839	0.003468326	0.030302214	Biological Process
GO:0090304	nucleic acid metabolic process	35	1660	0.003675795	0.0305091	Biological Process
GO:0009058	biosynthetic process	108	6647	0.00396414	0.031335585	Biological Process
GO:0007059	chromosome segregation	10	285	0.004662427	0.034278828	Biological Process
GO:0009987	cellular process	216	15282	0.004749476	0.034278828	Biological Process
GO:0044767	single-organism developmental process	78	4553	0.005172656	0.035777534	Biological Process
GO:0048869	cellular developmental process	72	4153	0.00570779	0.037899724	Biological Process
GO:0032502	developmental process	94	5732	0.006674087	0.042611481	Biological Process
GO:0030154	cell differentiation	68	3914	0.007120002	0.043774826	Biological Process
GO:0034641	cellular nitrogen compound metabolic process	110	6955	0.008993585	0.044377109	Biological Process
GO:0050896	response to stimulus	126	8146	0.009049023	0.044377109	Biological Process
GO:0009653	anatomical structure morphogenesis	41	2149	0.009402514	0.044377109	Biological Process
GO:0000278	mitotic cell cycle	12	419	0.009891283	0.044377109	Biological Process

GO:0000280	nuclear division	12	419	0.009891283	0.044377109	Biological Process
GO:0007067	mitotic nuclear division	12	419	0.009891283	0.044377109	Biological Process
GO:0022402	cell cycle process	12	419	0.009891283	0.044377109	Biological Process
GO:0048285	organelle fission	12	419	0.009891283	0.044377109	Biological Process
GO:1902589	single-organism organelle organization	12	419	0.009891283	0.044377109	Biological Process
GO:1903047	mitotic cell cycle process	12	419	0.009891283	0.044377109	Biological Process
GO:0061024	membrane organization	24	1100	0.0106238	0.046409232	Biological Process

Supplementary Table 4. Candidate non-coding host genes.

Host Gene ID	Description	Symbol	Type	Unique peptide coding potential
ENSG00000237298	TTN antisense RNA 1 [Source:HGNC Symbol;Acc:HGNC:44124]	TTN-AS1	antisense	yes
ENSG00000231721	long intergenic non-protein coding RNA, p53 induced transcript [Source:HGNC Symbol;Acc:HGNC:26885]	LINC-PINT	antisense	yes
ENSG00000240498	CDKN2B antisense RNA 1 [Source:HGNC Symbol;Acc:HGNC:34341]	CDKN2B-AS1	antisense	yes
ENSG00000264235		AP005329.1	antisense	no
ENSG00000281508	cerebellar degeneration related protein 1 [Source:HGNC Symbol;Acc:HGNC:1798]	CDR1	antisense	no
ENSG00000204625	HLA complex group 9 (non-protein coding) [Source:HGNC Symbol;Acc:HGNC:21243]	HCG9	lincRNA	no
ENSG00000256166		AL671883.2	lincRNA	no
ENSG00000251562	metastasis associated lung adenocarcinoma transcript 1 (non-protein coding) [Source:HGNC Symbol;Acc:HGNC:29665]	MALAT1	lincRNA	no
ENSG00000213468	firre intergenic repeating RNA element [Source:HGNC Symbol;Acc:HGNC:49627]	FIRRE	lincRNA	yes
ENSG00000229807	X inactive specific transcript (non-protein coding) [Source:HGNC Symbol;Acc:HGNC:12810]	XIST	lincRNA	yes

Supplementary Table 5. Primers and oligos used in this research.

PCR primers name	Forward primer（5‘to 3’）	Reverse primer（5‘to 3’）	Amplified product （bps）
QPCR-ciR-PINT	GCGTTCAGCCCTGGGGTCATAT	CAGTTTTTCTCTTCCGCAGCTA	106
QPCR-line-PINT	GGCTTGGCTAGTTGGAGAGTTAC	AACTGAAACCAGACCTAAGGTTTTG	112
QPCR-beta-actin	ACAGAGCCTCGCCTTTGCCGAT	CTTGCACATGCCGGAGCCGTT	109
Actin（Divergent primer）	GCACACCTTAAAAATGAGGCG	GGGTACTTCAGGGTGAGGATG	103
Actin（convergent primer）	TACGCCTCTGGCCGTACCAC	CCAGGTCCAGACGCAGGATG	115
PINT（Divergent primer）	AGGAACGAGGCAAGGAGCTA	TGCAGGAGGCAAAATGCAAT	156
convergent primer new	GGGCTTGGCAGCAGAAGGGA	AGGTACGGAGATATGACCTCTC	102
**FISH oligo sequences**			
FISH-ciR-PINT oligo	5‘Cy3-GAGATATGACCCCAGGGCTGAACC 3’		
FISH-ciR-PINT oligo-2	5' Cy3-GAAGTGAGGTACGGAGATATGACCCCAGGGCTGAACGCACGCT 3'		
FISH-line-PINT oligo	5‘FITC-TTCTTCCATTTTCCTCTCAGCTGTTACAGGGT3’		
**SiRNA sequences**			
**Name**	**Sense（5'-3'）**	**Antisense（5'-3'）**	
siRNA-ciR-PINT-1	AGCCCUGGGGUCAUAUCUCTT	GAGAUAUGACCCCAGGGCUTT	
siRNA-ciR-PINT-2	UUCAGCCCUGGGGUCAUAUTT	AUAUGACCCCAGGGCUGAATT	
siRNA-NC1	UGCGCUGCGCACAAAUGUGTT	CACAUUUGUGCGCAGCGCATT	
siRNA-NC2	AUCUGGCGUGCGCUCUUAATT	UUAAGAGCGCACGCCAGAUTT	
	**Upstream primer**	**Down-stream primer**	
q-TP53	CTGTGACTTGCACGTACTCC	CTGTGACTGCTTGTAGATG	
q-IKBKE	TGAACCACCAGAACATTG	AGCACCACCAGGAACTCATC	
q-DGKA	AGGATGGCGAGATGGCTAA	TTGAAACAGTGCCAGGCTT	
q-ADAM8	CCGCTACGTGGAGCTGTATG	GACCACACGGAAGTTGAGTT	
q-SERPINE2	ACCATAGACAGCTGGATGAG	TGCAGTTGTTGCTGCTGAAG	
oligo- gRNA87aa	CACCGCGCTGCAGGAGCCCCGATCG	AAACCGATCGGGGCTCCTGCAGCGC	
oligo- gRNA-NC	CACCGGCGCCTTAAGAGTACTCATC	AAACGATGAGTACTCTTAAGGCGCC	
gRNA-vector-hU6-sequencing	GAGGGCCTATTTCCCATGATT		
87aa- sequencing	TTGAGGACGCTCCTTCTGTT	ACTTGTCACGCCAGGCTGCT	
ALU gRNA-1	ACGGTGTTGTTCTGTCACCA		
ALU gRNA-2	TTTGTGTTTTTAGTAGGGGT		
GRNA0-F	5‘CACCGCTGAATCCAAGTAGTCCCAT 3’		
GRNA0-R	5‘AAACATGGGACTACTTGGATTCAGC3’		

F ALU 1-3	5' CGATTGGACGGTGTGAACTT 3'		
R ALU 1-3	5' TCCAACCACTCCACTGATGA 3'		
pa-1	CCAGTGTGGTAGCCACTAGC		
pa-2	GTGCCATCCAATACAGTAGC		
oligo-GRNA-alu-F1	CACCGACGGTGTTGTTCTGTCACCA	AAACTGGTGACAGAACAACACCGT	
oligo-GRNA-alu-F2	CACCGTTTGTGTTTTTAGTAGGGGT	AAACACCCCTACTAAAAACACAAAC	
LINC-PINT ASO-1	GTACGGAGATATGACCTCTCTTTA		
LINC-PINT ASO-2	GAGATATGACCTCTCTTTACCTGAA		
ASO-NC	TTGAGCGATATTCGACCTTACGTA		
q-TGFB1	CGTCTGCTGAGGCTCAAGTTA	CACAACTCCGGTGACATCAA	
q-NKX2-9	CGTGCGCAGCCTTCTAGAT	GCGCCTTGGAGAATAGCACC	
q-IL1R1	GAACAAGCCTCCAGGATTCA	TAAGTTAGGCTCATTCTCCACAA	
q-EGR2	CCACGTCGGTGACCATCTT	GAGATCCAACGACCTCTTCTC	
q-CPEB1	TCAGACACCAGTGGCTTCAG	GCAGCTTGCTCTTGGTCCAT	
CPEB1-pro-F	CGCGAGGGGGCGGAGGCGA		
CPEB1-pro-R	CCGGCAGCTTATGAAGCTCC		
CMYC-pro-F	AACAGGCAGACACATCTCAG		
CMYC-pro-R	AACGATGCCTAGAATGATTAAA		
SOX2-pro-F	GCGCTGATTGGTCGCTAGAA		
SOX2-pro-R	CAGCAAACTACTTTCCCCCTT		
Cycin D1-pro-F	CCTCCCGCTCCCATTCTCTG		
Cycin D1-pro-R	CTGGGGAGGGCTGTGGGTCCTG		
