# Supplementary material for: A peptide encoded by circular form of LINC-PINT suppresses oncogenic transcriptional elongation in glioblastoma
Source: Nat Commun. 2018 Oct 26;9:4475. doi: 10.1038/s41467-018-06862-2 (PMC6203777; doi:10.1038/s41467-018-06862-2)
Supplement: Supplementary file 4 — Supplementary Data 1 [file 41467_2018_6862_MOESM4_ESM.pdf]

Supplementary Data 1. Identified circRNAs through RNA-seq and RNC-seq.

ID	Source_Gene	Chr	Strand	Henomic_start	Henomic_end	Spliced_length	CircBase ID
novel_circ_000147	ENSG00000065526	1	+	15872816	15876678	1692	hsa_circ_0002237
novel_circ_000180	ENSG00000127483	1	-	20744744	20780540	3561	novel
novel_circ_000186	ENSG00000127483	1	-	20770930	20773610	304	hsa_circ_0005782
novel_circ_000480	ENSG00000187147	1	+	44411981	44412722	742	hsa_circ_0012152
novel_circ_000524	ENSG00000185104	1	-	50567077	50596216	524	hsa_circ_0008197
novel_circ_000533	ENSG00000085832	1	-	51402435	51408332	607	hsa_circ_0005567
novel_circ_000539	ENSG00000078618	1	-	51827796	51834170	228	hsa_circ_0009076
novel_circ_000553	ENSG00000203995	1	+	52877682	52892975	756	hsa_circ_0006685
novel_circ_000568	ENSG00000162600	1	-	58506060	58536741	865	hsa_circ_001068
novel_circ_000570	ENSG00000162600	1	-	58506060	58539310	1381	hsa_circ_0002316
novel_circ_000572	ENSG00000162600	1	-	58527261	58539310	1231	hsa_circ_0012721
novel_circ_000577	ENSG00000172456	1	+	59321536	59346398	479	hsa_circ_002012
novel_circ_000607	ENSG00000125703	1	+	62803719	62841547	1421	hsa_circ_0012824
novel_circ_000677	ENSG00000180488	1	+	77801331	77815231	700	hsa_circ_0002570
novel_circ_000688	ENSG00000117114	1	+	81836885	81907230	387	hsa_circ_0013048
novel_circ_000697	ENSG00000171517	1	-	84865385	84866138	754	hsa_circ_0004390
novel_circ_000734	ENSG00000162669	1	-	91343430	91385834	1841	novel
novel_circ_000764	ENSG00000067334	1	-	93876268	93877862	1595	hsa_circ_0013218
novel_circ_000846	ENSG00000224699	1	-	110359584	110360224	641	novel
novel_circ_000913	ENSG00000198162	1	+	117402186	117420649	553	hsa_circ_002173
novel_circ_000915	ENSG00000198162	1	+	117402186	117466427	866	hsa_circ_001022
novel_circ_001255	ENSG00000198700	1	+	201852104	201858994	954	novel
novel_circ_001288	ENSG00000158711	1	-	205616478	205623891	2071	hsa_circ_001035
novel_circ_001323	ENSG00000162769	1	+	212863725	212885396	458	hsa_circ_001674
novel_circ_001327	ENSG00000136643	1	+	213077696	213117410	331	hsa_circ_0004849
novel_circ_001360	ENSG00000143353	1	+	219193082	219241308	30103	hsa_circ_0004417
novel_circ_001401	ENSG00000185842	1	+	224952670	224968874	1045	hsa_circ_0016599
novel_circ_001402	ENSG00000185842	1	+	224952670	224974153	1108	hsa_circ_0016600
novel_circ_001403	ENSG00000185842	1	+	224952670	225007544	1385	hsa_circ_0016601
novel_circ_001404	ENSG00000185842	1	+	224952670	225023865	1636	novel

novel_circ_001412	ENSG00000185842	1	+	225002783	225051795	2333	novel
novel_circ_001630	ENSG00000179397	1	+	244552322	244561145	971	hsa_circ_0006080
novel_circ_001662	ENSG00000196418	1	-	247156406	247159813	1186	novel
novel_circ_007471	ENSG00000138031	2	+	24823978	24824617	640	novel
novel_circ_007569	ENSG00000115760	2	+	32488588	32493667	500	novel
novel_circ_007596	ENSG00000150938	2	+	36396614	36442735	538	hsa_circ_0002346
novel_circ_007674	ENSG00000068784	2	-	45546732	45553730	465	hsa_circ_0005542
novel_circ_007719	ENSG00000115310	2	-	54982515	54987698	347	hsa_circ_001289
novel_circ_007746	ENSG00000115464	2	-	61278165	61281242	435	hsa_circ_0007493
novel_circ_007785	ENSG00000082898	2	-	61522611	61526521	175	hsa_circ_001767
novel_circ_007813	ENSG00000119844	2	+	64551443	64553409	1967	hsa_circ_000026*
novel_circ_008016	ENSG00000115109	2	+	120127688	120175000	958	hsa_circ_0006834
novel_circ_008017	ENSG00000115109	2	+	120127688	120175004	962	hsa_circ_0008278
novel_circ_008088	ENSG00000121989	2	+	147896301	147899898	473	hsa_circ_000913
novel_circ_008142	ENSG00000198648	2	-	168138088	168182090	766	hsa_circ_0002029
novel_circ_008149	ENSG00000138399	2	-	169544721	169557297	845	hsa_circ_002052
novel_circ_008204	ENSG00000237298	2	+	178535732	178542361	2996	hsa_circ_0004305
novel_circ_008244	ENSG00000151690	2	+	190436528	190436677	150	novel
novel_circ_008358	ENSG00000138376	2	-	214752447	214792445	1462	hsa_circ_0058048
novel_circ_008421	ENSG00000135919	2	-	223998115	224001922	510	hsa_circ_001588
novel_circ_008428	ENSG00000144468	2	+	226864604	226867318	656	hsa_circ_0058493
novel_circ_008430	ENSG00000144468	2	+	226864604	226914351	1027	hsa_circ_0058495
novel_circ_008437	ENSG00000153827	2	-	229858772	229880128	1076	hsa_circ_0003273
novel_circ_009415	ENSG00000197548	3	+	11358418	11426926	797	hsa_circ_0008210
novel_circ_009459	ENSG00000154814	3	+	16294856	16303592	679	novel
novel_circ_009514	ENSG00000163527	3	+	31576396	31600459	563	hsa_circ_0003338
novel_circ_009630	ENSG00000173473	3	-	47610066	47678311	1586	hsa_circ_0065244
novel_circ_009698	ENSG00000004534	3	+	50065031	50066502	357	hsa_circ_0006531
novel_circ_009830	ENSG00000169855	3	-	78938601	78938927	327	hsa_circ_0004788
novel_circ_009841	ENSG00000080822	3	-	98515597	98515848	252	novel
novel_circ_009861	ENSG00000066422	3	-	101656104	101672213	3014	novel
novel_circ_009889	ENSG00000163611	3	-	113488945	113506605	611	hsa_circ_0006163

novel_circ_009900	ENSG00000181722	3	-	114350274	114351878	1605	hsa_circ_0005332
novel_circ_009971	ENSG00000058262	3	+	128060102	128060661	264	hsa_circ_0007841
novel_circ_009975	ENSG00000175792	3	-	128082483	128087798	185	hsa_circ_000694
novel_circ_010031	ENSG00000163785	3	-	134158121	134195182	1077	hsa_circ_0003113
novel_circ_010107	ENSG00000144935	3	+	142736379	142748460	460	hsa_circ_000697
novel_circ_010109	ENSG00000144935	3	+	142736379	142793960	2402	novel
novel_circ_010125	ENSG00000018408	3	-	149518751	149518950	200	novel
novel_circ_010153	ENSG00000169359	3	-	155833468	155842619	491	hsa_circ_0067774
novel_circ_010238	ENSG00000114450	3	-	179413412	179419505	603	hsa_circ_002097
novel_circ_010249	ENSG00000181449	3	+	181699598	181715292	15695	novel
novel_circ_010255	ENSG00000053524	3	-	183205876	183207823	388	novel
novel_circ_010341	ENSG00000119231	3	+	196885151	196886694	1544	hsa_circ_0068669
novel_circ_010373	ENSG00000159692	4	-	1225360	1241519	4198	hsa_circ_001895
novel_circ_010393	ENSG00000109685	4	+	1900626	1930770	1646	hsa_circ_0002688
novel_circ_010396	ENSG00000109685	4	+	1900626	1939778	1972	hsa_circ_0005881
novel_circ_010398	ENSG00000109685	4	+	1900626	1951203	11144	hsa_circ_001298
novel_circ_010448	ENSG00000132405	4	+	6923373	6924111	739	hsa_circ_000388
novel_circ_010449	ENSG00000132405	4	+	6923373	6967424	979	hsa_circ_0069101
novel_circ_010537	ENSG00000163697	4	-	41013583	41014398	816	hsa_circ_0005991
novel_circ_010602	ENSG00000150471	4	+	61934840	61998265	1283	novel
novel_circ_010647	ENSG00000138750	4	-	76134175	76144473	662	hsa_circ_0070039
novel_circ_010666	ENSG00000138756	4	+	78826037	78851056	705	hsa_circ_0070190
novel_circ_010709	ENSG00000145332	4	-	87163558	87195690	1975	novel
novel_circ_010760	ENSG00000168769	4	+	105233897	105237351	3455	hsa_circ_0070562
novel_circ_010829	ENSG00000145375	4	+	122927634	122979386	1706	novel
novel_circ_010907	ENSG00000151623	4	-	148435104	148436862	1759	hsa_circ_0071127
novel_circ_010993	ENSG00000109762	4	+	185247294	185267155	662	hsa_circ_0004874
novel_circ_011010	ENSG00000112877	5	+	618990	620261	321	hsa_circ_0071653
novel_circ_011013	ENSG00000112877	5	+	618990	655584	4293	novel
novel_circ_011031	ENSG00000112941	5	+	6737510	6746427	743	hsa_circ_0008903
novel_circ_011062	ENSG00000038382	5	+	14290716	14336727	1506	hsa_circ_0005683
novel_circ_011088	ENSG00000113384	5	-	32106124	32143880	20760	novel

novel_circ_011089	ENSG00000113384	5	-	32121495	32143880	5389	novel
novel_circ_011092	ENSG00000113384	5	-	32135572	32143880	247	hsa_circ_000725
novel_circ_011110	ENSG00000164190	5	+	36982165	36986301	4137	novel
novel_circ_011141	ENSG00000132357	5	+	40852174	40854096	1923	hsa_circ_0005895
novel_circ_011165	ENSG00000170571	5	-	50399107	50411383	954	hsa_circ_001310
novel_circ_011208	ENSG00000130449	5	+	61472681	61473037	357	hsa_circ_0005399
novel_circ_011224	ENSG00000113593	5	+	65567513	65572286	850	hsa_circ_0072697
novel_circ_011233	ENSG00000112851	5	+	66053406	66054951	1546	hsa_circ_0072732
novel_circ_011243	ENSG00000134057	5	+	69174877	69175537	378	hsa_circ_000024*
novel_circ_011255	ENSG00000145734	5	+	71502599	71512428	2199	novel
novel_circ_011295	ENSG00000171617	5	-	74634684	74636498	1815	hsa_circ_0006859
novel_circ_011305	ENSG00000198780	5	-	74834426	74841679	493	hsa_circ_0004405
novel_circ_011315	ENSG00000164252	5	+	77046347	77048272	443	hsa_circ_0008620
novel_circ_011404	ENSG00000133302	5	+	94643273	94654752	724	novel
novel_circ_011438	ENSG00000151422	5	+	108883396	108959347	928	hsa_circ_0006425
novel_circ_011541	ENSG00000113575	5	-	134199086	134206131	755	hsa_circ_0002602
novel_circ_011576	ENSG00000031003	5	-	137942875	137949184	658	novel
novel_circ_011578	ENSG00000031003	5	-	137985257	137988315	331	hsa_circ_002110
novel_circ_011662	ENSG00000113580	5	-	143399656	143400852	1197	hsa_circ_000420
novel_circ_011731	ENSG00000082515	5	+	154950821	154967237	884	novel
novel_circ_011759	ENSG00000113643	5	+	168488602	168497348	777	hsa_circ_001945
novel_circ_011763	ENSG00000113643	5	+	168500591	168518062	1051	hsa_circ_0074883
novel_circ_011848	ENSG00000127022	5	+	179709873	179720560	756	hsa_circ_0002051
novel_circ_011862	ENSG00000050748	5	-	180261684	180280608	738	hsa_circ_001168
novel_circ_011866	ENSG00000131459	5	-	180316341	180336577	1253	novel
novel_circ_011902	ENSG00000137267	6	-	3157407	3179712	22306	novel
novel_circ_011945	ENSG00000010017	6	-	13639563	13644729	598	hsa_circ_001173
novel_circ_011981	ENSG00000145996	6	+	20781145	20846178	225	hsa_circ_0008086
novel_circ_012048	ENSG00000206503	6	+	29891004	29945802	52219	novel
novel_circ_012052	ENSG00000204625	6	-	29944257	30008321	64065	novel
novel_circ_012074	ENSG00000256166	6	+	31268940	31354060	85121	novel
novel_circ_012075	ENSG00000256166	6	+	31268943	31354063	85121	novel

novel_circ_012076	ENSG00000256166	6	-	31268996	31354117	77923	novel
novel_circ_012077	ENSG00000256166	6	+	31268996	31354117	85122	novel
novel_circ_012078	ENSG00000256166	6	+	31269007	31354128	85122	novel
novel_circ_012079	ENSG00000256166	6	-	31269007	31354128	77923	novel
novel_circ_012084	ENSG00000256166	6	+	31270386	31355492	85107	novel
novel_circ_012256	ENSG00000112701	6	+	75634707	75678927	1722	hsa_circ_000747
novel_circ_012300	ENSG00000135315	6	-	84185187	84186623	554	hsa_circ_0077223
novel_circ_012315	ENSG00000188994	6	+	87215903	87218731	370	hsa_circ_0004058
novel_circ_012472	ENSG00000146350	6	-	121279121	121321794	1578	novel
novel_circ_012536	ENSG00000197442	6	-	136669283	136698682	754	hsa_circ_0009034
novel_circ_012591	ENSG00000055208	6	+	149378018	149379518	1501	hsa_circ_0078201
novel_circ_012640	ENSG00000213079	6	+	154773989	154833409	3800	novel
novel_circ_012662	ENSG00000130338	6	+	158282263	158314268	32006	novel
novel_circ_012704	ENSG00000112531	6	+	163455279	163478896	260	hsa_circ_0005328
novel_circ_012738	ENSG00000112584	6	+	170317370	170323259	1936	hsa_circ_0078768
novel_circ_012853	ENSG00000136237	7	-	22308339	22318037	449	novel
novel_circ_012891	ENSG00000106049	7	-	27629371	27632445	232	hsa_circ_0003958
novel_circ_012932	ENSG00000122507	7	+	33146242	33177591	572	hsa_circ_0003162
novel_circ_013002	ENSG00000179869	7	+	48219354	48249351	1718	novel
novel_circ_013016	ENSG00000106070	7	-	50669722	50756056	1250	novel
novel_circ_013089	ENSG00000075223	7	-	80789306	80810701	907	hsa_circ_0004365
novel_circ_013090	ENSG00000075223	7	-	80789306	80828745	1251	hsa_circ_0003634
novel_circ_013099	ENSG00000075213	7	-	84110470	84194668	535	hsa_circ_0004363
novel_circ_013125	ENSG00000001629	7	+	92294889	92327900	877	novel
novel_circ_013159	ENSG00000164715	7	+	98191614	98194572	2959	hsa_circ_0081207
novel_circ_012805	ENSG00000231721	7	-	131107720	131108803	1084	hsa_circ_0082389
novel_circ_013348	ENSG00000131558	7	+	133317284	133375002	526	hsa_circ_0008419
novel_circ_013350	ENSG00000198074	7	-	134527808	134534149	6342	novel
novel_circ_013353	ENSG00000227471	7	+	134533004	134569529	16827	novel
novel_circ_013370	ENSG00000155561	7	+	135577796	135593192	1182	hsa_circ_000751
novel_circ_013388	ENSG00000105894	7	-	137251230	137254974	452	hsa_circ_0003949
novel_circ_013458	ENSG00000146966	7	-	140601403	140602542	1140	hsa_circ_0002142

novel_circ_013491	ENSG00000106462	7	-	148846470	148847305	253	hsa_circ_0006357
novel_circ_013575	ENSG00000117868	7	-	158759486	158764853	495	hsa_circ_002142
novel_circ_013576	ENSG00000126870	7	+	158911550	158918869	461	hsa_circ_0083234
novel_circ_013599	ENSG00000104723	8	+	15650697	15673836	490	hsa_circ_0006410
novel_circ_013605	ENSG00000129422	8	-	17743604	17755961	2441	hsa_circ_0083444
novel_circ_013626	ENSG00000120910	8	+	22474954	22475624	323	hsa_circ_0083619
novel_circ_013674	ENSG00000156675	8	-	37870420	37877551	3262	hsa_circ_0005630
novel_circ_013706	ENSG00000083168	8	-	42048378	42049707	1057	hsa_circ_0002754
novel_circ_013818	ENSG00000140396	8	-	70159505	70162854	292	hsa_circ_001781
novel_circ_013833	ENSG00000147586	8	-	79917978	79919148	1171	novel
novel_circ_013933	ENSG00000164933	8	-	103404776	103407784	237	hsa_circ_0005499
novel_circ_013949	ENSG00000104447	8	-	115587001	115623758	2831	hsa_circ_0085362
novel_circ_013952	ENSG00000104447	8	-	115619132	115623758	1097	hsa_circ_0006950
novel_circ_014028	ENSG00000153317	8	-	130358017	130361771	229	hsa_circ_0085616
novel_circ_014102	ENSG00000107249	9	-	4117768	4118881	1114	hsa_circ_0006370
novel_circ_014105	ENSG00000107249	9	-	4286038	4286523	486	hsa_circ_0002874
novel_circ_014168	ENSG00000240498	9	+	22046751	22097364	2094	hsa_circ_0008796
novel_circ_014192	NA	9	-	33624261	33624617	357	novel
novel_circ_014206	ENSG00000137073	9	-	33971651	34017189	720	hsa_circ_0086732
novel_circ_014222	ENSG00000198853	9	+	35546430	35548535	2106	hsa_circ_0002702
novel_circ_014273	ENSG00000107362	9	-	71866799	71875083	858	hsa_circ_0006383
novel_circ_014297	ENSG00000197969	9	+	77337255	77371149	4627	hsa_circ_0087255
novel_circ_014335	ENSG00000135049	9	-	85575315	85633374	2201	novel
novel_circ_014341	ENSG00000135049	9	-	85585463	85633374	1863	hsa_circ_0087386
novel_circ_014342	ENSG00000135049	9	-	85596362	85633374	1121	hsa_circ_0007162
novel_circ_014344	ENSG00000135049	9	-	85618983	85633374	1033	hsa_circ_000999
novel_circ_014377	ENSG00000048828	9	+	93471141	93498886	556	hsa_circ_001209
novel_circ_014433	ENSG00000136891	9	-	100303632	100349372	2685	novel
novel_circ_014439	ENSG00000241697	9	+	100498765	100516771	364	hsa_circ_0004425
novel_circ_014486	ENSG00000165181	9	+	111786793	111787947	1155	novel
novel_circ_014638	ENSG00000130723	9	+	131430090	131432794	348	hsa_circ_000760
novel_circ_001708	ENSG00000057608	10	-	5773842	5800705	774	hsa_circ_0017586

novel_circ_001709	ENSG00000057608	10	-	5785142	5800705	674	hsa_circ_0005379
novel_circ_001729	ENSG00000198879	10	-	7276892	7285954	2118	hsa_circ_000524
novel_circ_001732	ENSG00000198879	10	-	7367649	7381949	487	hsa_circ_0017648
novel_circ_001877	ENSG00000107643	10	+	48401612	48410168	499	hsa_circ_0002968
novel_circ_001888	ENSG00000108064	10	+	58398189	58400285	2097	novel
novel_circ_001910	ENSG00000148634	10	-	68025546	68044563	1233	hsa_circ_0007113
novel_circ_002105	ENSG00000077147	10	-	96544076	96576829	1083	novel
novel_circ_002180	ENSG00000108055	10	+	110596398	110598290	305	hsa_circ_002043
novel_circ_002196	ENSG00000107518	10	+	115120185	115129535	536	hsa_circ_0020093
novel_circ_002203	ENSG00000151892	10	-	116125221	116125557	337	hsa_circ_0020101
novel_circ_002240	ENSG00000182022	10	-	124038515	124046724	1702	hsa_circ_001901
novel_circ_002245	ENSG00000189319	10	-	124681607	124706887	1080	hsa_circ_0002282
novel_circ_002270	ENSG00000150760	10	+	126996748	127000307	512	hsa_circ_0003970
novel_circ_002309	ENSG00000078902	11	-	1286002	1295794	577	hsa_circ_0008301
novel_circ_002392	ENSG00000187079	11	+	12862250	12864900	128	hsa_circ_0006284
novel_circ_002419	ENSG00000129158	11	-	17988597	18008076	824	hsa_circ_0021412
novel_circ_002422	ENSG00000110756	11	-	18291442	18292019	578	hsa_circ_000109
novel_circ_002494	ENSG00000026508	11	+	35229142	35229309	168	novel
novel_circ_002497	ENSG00000179241	11	+	36227085	36227430	346	hsa_circ_0006988
novel_circ_002672	ENSG00000251562	11	+	65499721	65499847	127	novel
novel_circ_002754	ENSG00000131626	11	+	70330173	70335694	498	novel
novel_circ_002815	ENSG00000137513	11	-	78465876	78493195	475	hsa_circ_0005935
novel_circ_002868	ENSG00000184384	11	-	96091892	96093517	1626	hsa_circ_0024085
novel_circ_002916	ENSG00000149311	11	+	108315823	108335961	2262	hsa_circ_0024231
novel_circ_002988	ENSG00000154114	11	+	121035733	121060085	1772	novel
novel_circ_003023	ENSG00000134909	11	-	129123446	129164427	328	hsa_circ_0007843
novel_circ_003186	ENSG00000151746	12	+	32305697	32306122	426	hsa_circ_0025839
novel_circ_003202	ENSG00000134283	12	+	42351885	42398994	837	hsa_circ_0004815
novel_circ_003296	ENSG00000205426	12	-	52287984	52316593	26028	novel
novel_circ_003297	ENSG00000170523	12	-	52287984	52362979	69773	novel
novel_circ_003337	ENSG00000111602	12	-	56430881	56432524	378	hsa_circ_002140
novel_circ_003403	ENSG00000111605	12	+	69251129	69262562	1710	hsa_circ_001472

novel_circ_003409	ENSG00000166226	12	+	69589485	69592159	304	hsa_circ_001726
novel_circ_003469	ENSG00000111144	12	+	96035378	96036105	728	novel
novel_circ_003536	ENSG00000110880	12	-	108652272	108654410	251	hsa_circ_002167
novel_circ_003575	ENSG00000204842	12	-	111554158	111555919	97	hsa_circ_001229
novel_circ_003639	ENSG00000089154	12	-	120154970	120155719	389	hsa_circ_000153
novel_circ_003766	ENSG00000176915	12	-	132747832	132755133	1049	hsa_circ_0008567
novel_circ_003837	ENSG00000102781	13	-	30227412	30283791	1161	hsa_circ_0008068
novel_circ_003904	ENSG00000172766	13	-	41320825	41323042	2218	novel
novel_circ_004001	ENSG00000136111	13	-	75326197	75362607	1535	hsa_circ_0030427
novel_circ_004022	ENSG00000005810	13	-	77217840	77243951	676	hsa_circ_0004217
novel_circ_004029	ENSG00000139737	13	+	77719532	77753358	1723	hsa_circ_000839
novel_circ_004031	ENSG00000152193	13	-	78635110	78644997	566	hsa_circ_000840
novel_circ_004181	ENSG00000100473	14	+	30877572	30880734	547	hsa_circ_0031431
novel_circ_004213	ENSG00000129521	14	-	33925618	33931215	636	novel
novel_circ_004325	ENSG00000126773	14	+	60115719	60125823	1910	hsa_circ_000591
novel_circ_004504	ENSG00000100796	14	-	91481576	91485730	717	hsa_circ_0003045
novel_circ_004524	ENSG00000066739	14	-	96341522	96347341	762	hsa_circ_0002223
novel_circ_004532	ENSG00000100749	14	+	96833467	96860735	1073	hsa_circ_002156
novel_circ_004538	ENSG00000183576	14	-	99458279	99465813	683	hsa_circ_001983
novel_circ_004661	ENSG00000174197	15	+	41668828	41669958	1131	hsa_circ_000021
novel_circ_004668	ENSG00000174197	15	+	41696075	41699159	1124	hsa_circ_001251
novel_circ_004772	ENSG00000069974	15	-	55223889	55234956	489	hsa_circ_0003863
novel_circ_004872	ENSG00000103591	15	-	67231814	67236820	462	hsa_circ_000603
novel_circ_004912	ENSG00000066933	15	-	72032494	72046634	1006	hsa_circ_0002506
novel_circ_004913	ENSG00000066933	15	-	72045724	72046634	911	hsa_circ_0006509
novel_circ_004962	ENSG00000140391	15	-	77046776	77056255	758	novel
novel_circ_004967	ENSG00000173517	15	-	77178790	77182040	3251	hsa_circ_0036423
novel_circ_004982	ENSG00000169330	15	+	79456098	79458445	2348	novel
novel_circ_004983	ENSG00000169330	15	+	79456098	79469145	3562	novel
novel_circ_004985	ENSG00000086666	15	+	80098416	80122800	799	hsa_circ_002031
novel_circ_005003	ENSG00000073417	15	+	85113873	85115487	214	hsa_circ_0003856
novel_circ_005043	ENSG00000140575	15	+	90441506	90443478	264	hsa_circ_000861

novel_circ_005075	ENSG00000140443	15	+	98707562	98708107	546	hsa_circ_0005035
novel_circ_005114	ENSG00000103227	16	-	910931	954666	773	hsa_circ_000621
novel_circ_005245	ENSG00000103222	16	+	16141173	16142942	1770	novel
novel_circ_005337	ENSG00000080603	16	+	30728966	30729572	469	hsa_circ_0006127
novel_circ_005350	ENSG00000197302	16	+	31722626	31723353	223	hsa_circ_0007059
novel_circ_005362	ENSG00000171208	16	-	47109483	47132025	849	hsa_circ_0003520
novel_circ_005393	ENSG00000177200	16	+	53141179	53157541	16363	novel
novel_circ_005111	ENSG00000039068	16	+	68811684	68815759	733	hsa_circ_0039992
novel_circ_005510	ENSG00000132604	16	-	69370483	69372355	234	novel
novel_circ_005668	ENSG00000176715	16	+	89098591	89102759	1064	hsa_circ_0006958
novel_circ_005820	ENSG00000133026	17	-	8569720	8577338	226	hsa_circ_0042049
novel_circ_005885	ENSG00000072210	17	+	19651547	19671956	1566	hsa_circ_0008603
novel_circ_005901	ENSG00000128487	17	+	20204333	20205912	1580	hsa_circ_000013
novel_circ_005917	ENSG00000087095	17	+	28122603	28172618	691	hsa_circ_0002103
novel_circ_006285	ENSG00000154240	17	-	66175044	66183307	927	novel
novel_circ_006340	ENSG00000184640	17	+	77402059	77402703	645	hsa_circ_0005320
novel_circ_006445	ENSG00000132205	18	+	2890561	2892486	1926	hsa_circ_0004658
novel_circ_006448	ENSG00000264235	18	-	3255839	3277857	17070	novel
novel_circ_006491	ENSG00000101639	18	+	12999421	13019206	1054	hsa_circ_001089
novel_circ_006495	ENSG00000101639	18	+	12999421	13042334	2071	hsa_circ_0046999
novel_circ_006531	ENSG00000101752	18	+	21765772	21779685	679	hsa_circ_002013
novel_circ_006586	ENSG00000134758	18	+	32111754	32113860	282	hsa_circ_0005729
novel_circ_006632	ENSG00000175387	18	-	47865059	47896809	785	hsa_circ_000640
novel_circ_006662	ENSG00000198796	18	-	58578814	58580548	1735	novel
novel_circ_006692	ENSG00000101493	18	-	76441245	76443211	1967	hsa_circ_0008699
novel_circ_006696	ENSG00000130856	18	+	76849526	76871825	612	hsa_circ_0001993
novel_circ_006698	ENSG00000130856	18	+	76849526	76881512	1362	hsa_circ_000642
novel_circ_006825	ENSG00000167670	19	+	4408903	4409759	857	hsa_circ_0048607
novel_circ_006930	ENSG00000130164	19	+	11120092	11120522	295	hsa_circ_0006877
novel_circ_007005	ENSG00000130299	19	+	17332272	17339599	3989	novel
novel_circ_007017	ENSG00000105701	19	-	18537601	18538433	391	hsa_circ_0050119
novel_circ_007119	ENSG00000105221	19	-	40255158	40265351	401	hsa_circ_0008719

novel_circ_007188	ENSG00000160007	19	+	46918488	46922356	3869	novel
novel_circ_007189	ENSG00000160007	19	+	46918488	46937408	4014	hsa_circ_001101
novel_circ_007351	ENSG00000198131	19	+	58260851	58263676	2826	novel
novel_circ_008592	ENSG00000089006	20	-	17947486	17957037	1027	hsa_circ_0001977
novel_circ_008620	ENSG00000100994	20	+	25297080	25297394	315	novel
novel_circ_008634	ENSG00000171456	20	+	32366384	32369123	198	hsa_circ_002158
novel_circ_008679	ENSG00000214078	20	-	35653528	35659014	378	hsa_circ_000657
novel_circ_008684	ENSG00000131051	20	-	35714185	35725155	680	hsa_circ_0004870
novel_circ_008834	ENSG00000101190	20	-	62854016	62860308	733	hsa_circ_0004926
novel_circ_008879	ENSG00000156273	21	+	29305204	29329693	1955	novel
novel_circ_008889	ENSG00000156299	21	-	31164962	31203012	603	hsa_circ_0061405
novel_circ_009104	ENSG00000100068	22	-	25375813	25381578	394	hsa_circ_0003102
novel_circ_009237	ENSG00000100201	22	-	38494630	38501280	927	hsa_circ_0002211
novel_circ_009240	ENSG00000100206	22	-	38521608	38568289	1912	hsa_circ_001972
novel_circ_009322	ENSG00000100271	22	-	43039780	43046573	290	novel
novel_circ_014744	ENSG00000198786	MT	+	12430	12666	237	novel
novel_circ_014786	ENSG00000101871	X	-	10523092	10567603	812	hsa_circ_0007718
novel_circ_014788	ENSG00000101871	X	-	10566888	10567603	716	hsa_circ_0007933
novel_circ_014790	NA	X	+	13666317	13680598	14282	hsa_circ_0007717
novel_circ_014826	ENSG00000005889	X	+	24172715	24179770	791	hsa_circ_0007108
novel_circ_014889	ENSG00000086758	X	-	53645311	53654131	528	hsa_circ_002081
novel_circ_014971	ENSG00000229807	X	+	73846015	73846588	574	novel
novel_circ_014980	ENSG00000229807	X	+	73852031	73852274	244	novel
novel_circ_014986	ENSG00000229807	X	+	73852123	73852274	152	novel
novel_circ_014987	ENSG00000229807	X	+	73852123	73852319	197	novel
novel_circ_015027	ENSG00000133131	X	-	106980891	106985250	417	novel
novel_circ_015030	ENSG00000089682	X	-	107088436	107088839	404	hsa_circ_0002995
novel_circ_015032	ENSG00000080561	X	+	107840670	107854704	812	hsa_circ_0002153
novel_circ_015067	ENSG00000198918	X	-	119787284	119788271	988	novel
novel_circ_015101	ENSG00000213468	X	-	131743096	131794466	998	novel
novel_circ_015103	ENSG00000213468	X	-	131749306	131794466	845	hsa_circ_001992
novel_circ_015131	ENSG00000184258	X	-	140783165	140784649	1485	novel

novel_circ_015132	ENSG00000184258	X	-	140783170	140784654	1485	novel
novel_circ_015133	ENSG00000281508	X	+	140783175	140784659	1485	CDR1as
